# Time-Dependent Subcellular Distribution and Effects of Carbon Nanotubes in Lungs of Mice

**DOI:** 10.1371/journal.pone.0116481

**Published:** 2015-01-23

**Authors:** Carsten Købler, Sarah S. Poulsen, Anne T. Saber, Nicklas R. Jacobsen, Håkan Wallin, Carole L. Yauk, Sabina Halappanavar, Ulla Vogel, Klaus Qvortrup, Kristian Mølhave

**Affiliations:** 1 DTU Nanotech, Technical University of Denmark, Kgs. Lyngby, Denmark; 2 DTU CEN, Technical University of Denmark, Kgs. Lyngby, Denmark; 3 National Research Centre for the Working Environment, Copenhagen, Denmark; 4 Department of Science, Systems and Models, Roskilde University, Roskilde, Denmark; 5 Institute of Public Health, University of Copenhagen, Copenhagen, Denmark; 6 Environmental and Radiation Health Sciences Directorate, Health Canada, Ontario, Canada; 7 Department of Biomedical Sciences, CFIM, University of Copenhagen, Copenhagen, Denmark; Universidad de Castilla-La Mancha, SPAIN

## Abstract

**Background and Methods:**

Pulmonary deposited carbon nanotubes (CNTs) are cleared very slowly from the lung, but there is limited information on how CNTs interact with the lung tissue over time. To address this, three different multiwalled CNTs were intratracheally instilled into female C57BL/6 mice: one short (850 nm) and tangled, and two longer (4 μm and 5.7 μm) and thicker. We assessed the cellular interaction with these CNTs using transmission electron microscopy (TEM) 1, 3 and 28 days after instillation.

**Results:**

TEM analysis revealed that the three CNTs followed the same overall progression pattern over time. Initially, CNTs were taken up either by a diffusion mechanism or via endocytosis. Then CNTs were agglomerated in vesicles in macrophages. Lastly, at 28 days post-exposure, evidence suggesting CNT escape from vesicle enclosures were found. The longer and thicker CNTs more often perturbed and escaped vesicular enclosures in macrophages compared to the smaller CNTs. Bronchoalveolar lavage (BAL) showed that the CNT exposure induced both an eosinophil influx and also eosinophilic crystalline pneumonia.

**Conclusion:**

Two very different types of multiwalled CNTs had very similar pattern of cellular interactions in lung tissue, with the longer and thicker CNTs resulting in more severe effects in terms of eosinophil influx and incidence of eosinophilic crystalline pneumonia (ECP).

## Introduction

Carbon nanotubes (CNTs) have excellent mechanical and electrical properties and have therefore been of great interest to researchers since their discovery some 50 year ago [1]. The increasing number of possible applications for CNTs evokes an increasing need to investigate their toxicity. The possible asbestos-like toxicity of CNTs causes particular concern [2]. Inhaled CNTs are removed very slowly from the lung, and the half-life of CNTs in lung following inhalation in mice has been estimated to be ca. 1 year [3]. CNTs have been shown to cause pulmonary long-lasting inflammation, fibrosis and granulomas in lungs of rodents following pulmonary exposure [3–5].

Assessment of CNT-toxicity is complicated by the wide variety of physical and chemical properties with which they can be manufactured. For instance, the physical dimensions of CNTs have been reported to play an important role in their toxicity [6–8], and the length/width ratios of some CNTs are consistent with the fiber pathogenicity paradigm [2]. The number of defects in the lattice structure of CNTs also plays a role in their toxicity [9], together with agglomeration level [10] and functionalization [11–13]. In addition to the direct interaction between CNTs and cells, their toxicity is further complicated by the ability of CNTs to adsorb biomolecules and essentially transform into different biological identities [14].

Inhaled CNTs are met by a dynamic response responsible for clearing and dealing with particulates in the lung. The lung responds to CNTs by a rapid influx of neutrophils and macrophages [5,15,16], and in certain cases also by eosinophil influx [16–19]. Within a few days to weeks, exposure to even small doses of CNTs induces inflammation, granulomas and fibrosis [2,3,15]. CNT-cell interaction therefore changes over time, making it necessary to study the CNT-cell interaction at multiple time points.

In order to be able to visualize single CNTs inside cells, and investigate how the CNT-cell interaction changes over time, transmission electron microscopy (TEM) can be used. The time dependent effects of CNT exposure *in vivo* have mainly been studied using light microscopy, where electron microscopy is used to support the results [20–22]. TEM has, for example, revealed low CNT concentrations in neutrophils and type II pneumocytes, whereas high CNT-concentrations were readily visible in alveolar macrophages using light microscopy [20].

Cellular uptake and clearance mechanisms of CNTs have predominantly been studied *in vitro*. CNTs are taken up either as agglomerates entering the endosomal pathway by endocytosis, or as single CNTs piercing the cell membrane in what appears to be a non-endocytotic diffusion-driven process [7,12,23–25]. In addition, it has been argued that after direct non-endocytotic uptake, CNTs can subsequently be engulfed in vesicles [12,24], and that CNTs already contained in vesicles may escape into the cytosol [23,24]. Lastly, CNTs can escape the cell via microvesicles [26] or membrane disruption [12]. These *in vitro* studies illustrate the complexity of the interactions, the need for high resolution microscopy to understand the details of CNT toxicity and the need for *in vivo* studies to test if these conclusions hold in the presence of an active immune response.

Here, we study the ultra-structural time course of CNT distribution *in vivo* 1, 3 and 28 days after intratracheal instillation of CNTs in mice with a focus on TEM imaging. In addition, we document the differences and similarities in pulmonary toxicological response of mice exposed to three CNTs, of which two have very different physiochemical properties and two were very similar. Lung samples were imaged with transmission electron microscopy (TEM), and the results compared with bronchoalveolar lavage (BAL) cell composition and relevant gene expression data to investigate the induced eosinophilic crystalline pneumonia (ECP) [16].

## Materials and Methods

### CNTs

Three types of multiwalled CNTs (MWCNT) were used. A detailed description of the physiochemical data is found in S1 Table:

- NRCWE-026 (CNT_Small_) has an average length of 850 nm and width of 10 nm. It was used in a previous study [27].
- NM-401 (CNT_Large_) is on average 4 μm long and 70 nm wide. It was also used in a previous study [27] and was a test material in the Nanogenotox project [28].
- The last MWCNT (denoted Mitsui-7) has an average length of 5.7 μm and width of 75 nm [16,17,29]. It was also used in the Nanogenotox project, under the name NRCWE-006 [28].

### Mice

Female C57BL/6 mice, 5–7 weeks old, obtained from Taconic, were allowed water and food (Altromin # 1324) *ad libitum* during the experiment. The animals were acclimatised for two weeks and housed in experimental groups in polypropylene cages with sawdust. The environment was controlled with a temperature of 21 ± 1°C, a humidity of 50 ± 10% and a 12-h light/dark cycle. Experiments complied with EC Directive 86/609/EEC and were approved by the Danish “Animal Experiments Inspectorate” (permit 2010/561-1779).

### Exposure

Particle suspensions and intratracheal instillation were described previously [16,27]. Animals were exposed to 18, 54 or 162 μg/mouse, and respectively correspond to the pulmonary deposition of CNTs during 4, 11 or 32 eight-hour working days at the current Danish occupational exposure level for carbon black (3.5 mg/m^3^) assuming 10% pulmonary deposition [15].

This study was done with mice used for several ongoing studies and to limit the number of mice, there are minor variations in the instillation procedure, which does not seem to be influencing the results: Briefly, the Mitsui-7 CNTs were suspended in 0.9%wt NaCl and 10% v/v acellular bronchoalveolar lavage (BAL) fluid. CNT_Small_ and CNT_Large_ were suspended in 2% serum in Nanopure water. Serum and BAL fluid were obtained from unexposed C57BL/6 mice. The Mitsui-7 (4.05 mg/ml) and the CNT_Small_ and CNT_Large_ (3.24 mg/ml) suspensions were sonicated on ice using a Branson Sonifier S-450D equipped with a disruptor horn (Model number: 101-147-037). Mitsui-7 was sonicated for a total of 4 minutes at 10% amplitude, with alternating 10 s pulses and pauses, while CNT_Small_ and CNT_Large_ were sonicated for a total of 16 min at 400 W and 10% amplitude. These suspensions were used for the high dose (162 μg) and diluted 1:3 in vehicle for the medium (54 μg) dose and diluted further 1:3 for the low dose (18 μg). Between the dilutions the suspensions were mixed by pipetting. Vehicle control solutions were prepared with 0.9% NaCl and 10% acellular BAL fluid, and 2% serum in Nanopure water, respectively, and were sonicated as described above. Mice were intratracheally instilled with 40 μl (Mitsui-7) and 50 μL (CNT_Small_ and CNT_Large_) particle suspension, respectively. As discussed in the Results section, no discernible differences were observed between the two large CNT types in different suspension medium.

### Lung tissue

At 1, 3, and 28 days after the intratracheal instillation, the mice dedicated for electron microscopy were anaesthetized by subcutaneous injection of Hypnorm–Dormicum and the mice were bled by cutting the groin. The lungs were fixed *in situ* by cannulating the trachea and infusing 2% glutaraldehyde in 0.05 M cacodylate buffer (pH 7.2) at a constant fluid pressure of 30 cm before the thorax was opened. The lungs were excised and immersed in 2% glutaraldehyde 0.05 M cacodylate buffer (pH 7.2) and stored refrigerated until further processing.

### BAL cells composition

One, 3, and 28 days after intratracheal instillation, mice dedicated for the other endpoints (BAL cell counts and microarray gene expression on lung tissue) were anaesthetized by subcutaneous injection of Hypnorm-Dormicum. BAL fluid was collected directly after cardiac puncture and centrifuged. The cellular pellet was collected on glass slides and stained with standard May-Grünwald-Giemsa. The number of neutrophils, macrophages, eosinophils and lymphocytes cells were counted (n = 6). Lung tissue was snap frozen in liquid nitrogen and stored at −80°C until RNA purification. Statistical analysis was performed in SAS v. 9.2. A non-parametric one-way ANOVA test with a Tukey-type mean comparison was used to determine statistical significance.

### Gene expression

Total RNA (n = 6 per dose group) was extracted and isolated from the lung tissue of Mitsui-7 exposed mice (doses: 18, 54 and 162 μg on post-exposure day 1, and 54 μg on post-exposure day 28), from the CNT_Small_ and CNT_Large_ exposed mice (doses: 18, 54 and 162 μg on post-exposure day 1, 3 and 28), and from concurrent controls as previously described [16]. All samples passed quality control and were used in the microarray hybridization measurement. The microarray hybridization was performed on Agilent 8 × 60K oligonucleotide microarrays (Agilent Technologies Inc., Mississauga, ON, Canada) using 200 ng total RNA from each sample. Both the hybridization procedures and statistical analyses have previously been described [16]. Global gene expression results will be published elsewhere (Poulsen *et al*. in peer review), and here we report specifically mRNA levels of *Ccl11, Ccl24* and *Chi3L3*.

### Electron microscopy sample preparation

We investigated one CNT sample (164 μg) from each time point by electron microscopy. Approximately 1 mm^3^ samples of lung tissue were cut with a scalpel and embedded in Epon [27]. Briefly, the samples were rinsed in buffer and postfixed in 2% osmium tetroxide and 0.05 M potassium ferricyanide in 0.12 M sodium cacodylate buffer (pH 7.2) for 2 hours. Samples were then rinsed in ultrapure water, en-bloc stained with 1% uranyl acetate in water overnight, dehydrated in ethanol and embedded in epon following standard protocols for the TAAB 812 resin kit, TAAB Laboratories Equipment.

Ultramicrotomy of the samples was performed on a Leica Ultracut with a diamond-knife-angle of 6° and a cutting speed of 1.5 mm/s. Due to extensive microtomy artefacts caused by the hard CNTs [27], we used thicker TEM sections (approximately 200–300 nm) on selected CNT_Large_ and Mitsui-7 samples. TEM sections were post-stained with uranyl acetate and lead citrate (Ultrastain-2, Leica Microsystems), and imaged on a CM 100 BioTWIN (Philips) operated at 80 kV. For 3D imaging of ECP crystals, focused ion beam scanning electron microscopy (FIB-SEM) imaging was performed using a FEI QuantaFEG 3D with a dedicated vC backscattered electron detector operated at 3 kV and spot 1. Milling was performed on the ultramicrotomy prepared blocks and the images processed as previously described [27].

## Results

### BAL fluid cell composition

Intratracheal instillation of the three different CNTs resulted in pulmonary inflammation in terms of increased number of cells in BAL (S2 Table). In general, similar changes in BAL cell composition were observed for the three different CNT types as described previously [16] and (Poulsen et al. in peer review). The BAL cell influx was dominated by a large neutrophil influx that peaked on day 3 compared to vehicle-exposed controls.

Increased eosinophil counts were observed for all the CNT types on day 1 and 3, and eosinophil counts were higher for CNT_Large_ and Mitsui-7 compared to CNT_Small_ (Table 1). On day 28, only the eosinophil counts of Mitsui-7 were statistically significantly different from vehicle controls.

**Table 1:** Overview of *Chi3L3* mRNA levels, BAL eosinophil counts and presence of eosinophilic crystals in BAL cell fluid.

		**Day 1**		**Day 3**		**Day 28**		
	**Dose [μg]**	**Chi3L3**	**Eos (×10^3^)**	**Chi3L3**	**Eos (×10^3^)**	**Chi3L3**	**Eos (×10^3^)**	**ECP**
	0	-	1.0	-	3.9	-	10.5	1/12
								
**CNT_Small_**	18	2.0	17*	1.4	69*	1.0	0.5	2/6
	54	1.0	1.4*	0.5	72*	0.9	0.2	1/6
	162	1.0	3.4*	0.7	7.1	1.7	0.0	3/6
								
**CNT_Large_**	18	2.8*	51*	5.8*	317*	1.8	34	6/6
	54	3.1*	86*	4.3*	138*	2.5	32	5/6
	162	1.1	1.7	1.1	1.8	9.2*	46	4/6
								
**Mitsui-7**	0	-	0.3	-	0.4	-	0.3	1/6
	18	2.9*	39*	-	341*	-	5.5*	6/6
	54	1.8	23*	-	268*	3.9*	22*	6/6
	162	2.0	2.2*	-	102*	-	46*	6/6

*Chi3L3* expression is given as the relative fold-increase in *Chi3L3* mRNA levels relative to concurrent controls, where values below 1 indicate a decreased mRNA level. Eos denotes the number of eosinophils. The control groups for CNT_Small_ and CNT_Large_ were pooled, since these were instilled using the same instillation vehicle (2% serum) and BAL cell composition of the control groups was not significantly different from each other. Mitsui-7 was instilled using a different vehicle (10% BAL in 0.9%NaCl), and BAL cell composition of Mitsui-7 controls was statistically different from the other control groups. Asterisk (*) denotes data statistically significantly different from controls (p<0.05). ECP indicates how many of the samples (n = 6) contained eosinophilic crystals. Dashes (–) indicate no data available.

### Electron microscopy

The interactions between the three types of CNTs with lung tissue were studied over time using TEM. We compared CNT-tissue interactions of CNT_Small_ (850 nm long and 10 nm wide) and CNT_Large_ (4 μm long and 70 nm wide) for structurally induced differences.

**Figure 1:**
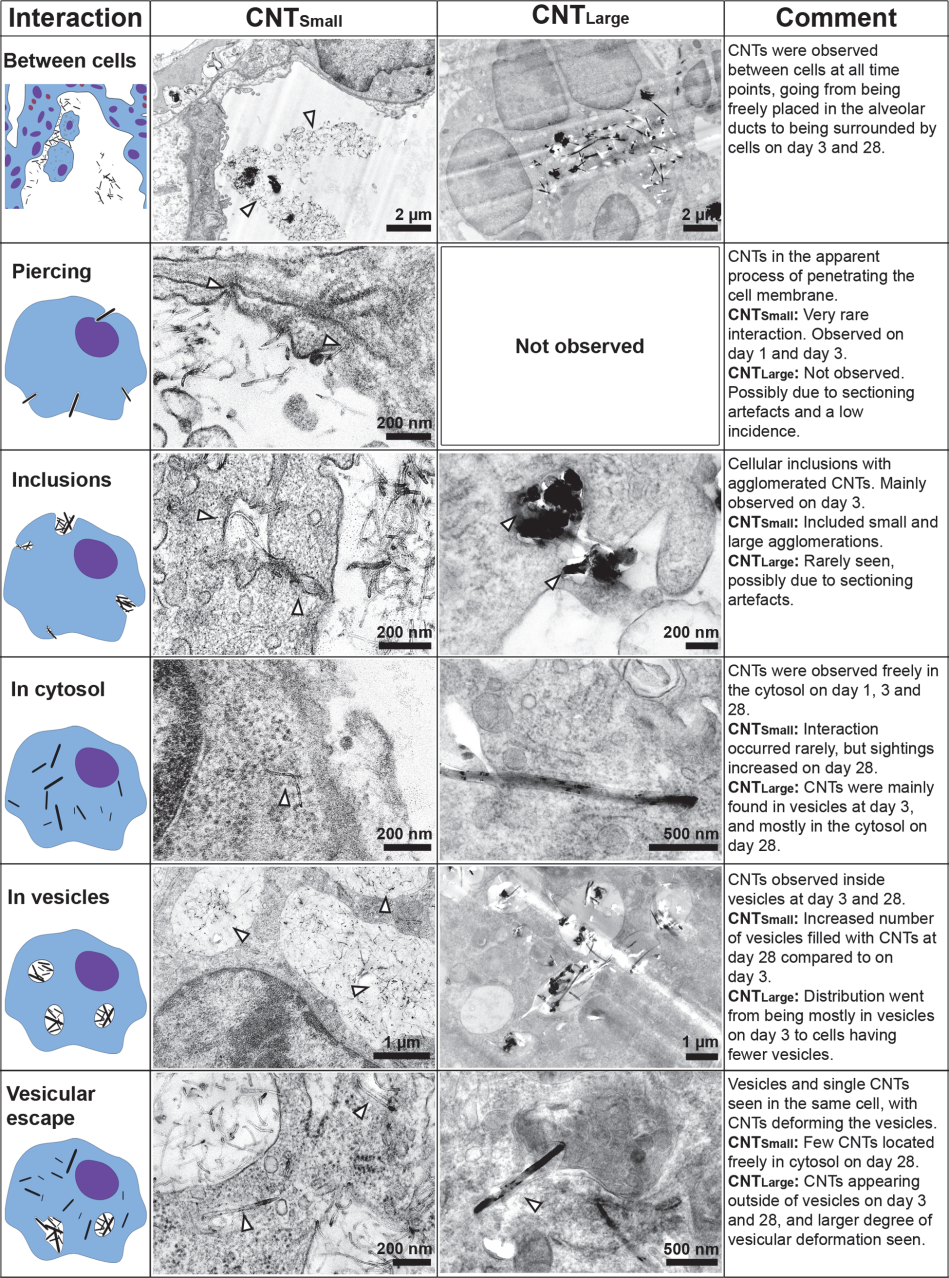
Overview of the observed CNT-cell interactions. Each interaction has a descriptive sketch, an example image of how the interaction was expressed in CNT_Small_ and CNT_Large_ exposed samples, and a description of the differences and progression of the interaction. White arrowheads indicate the presence of CNTs in TEM images.

The reproducibility of the interactions was evaluated by comparing CNT_Large_ and Mitsui-7 (5.7 μm long and 75 nm wide). TEM imaging showed that these two fairly similar tubes also showed similar CNT-cell interactions, and thus we only show images of CNT_Small_ and CNT_Large_ (Fig. 1). Lung tissue from vehicle-exposed mice revealed no CNT-like structures and there was no discernible difference between the different suspension media.

**Figure 2:**
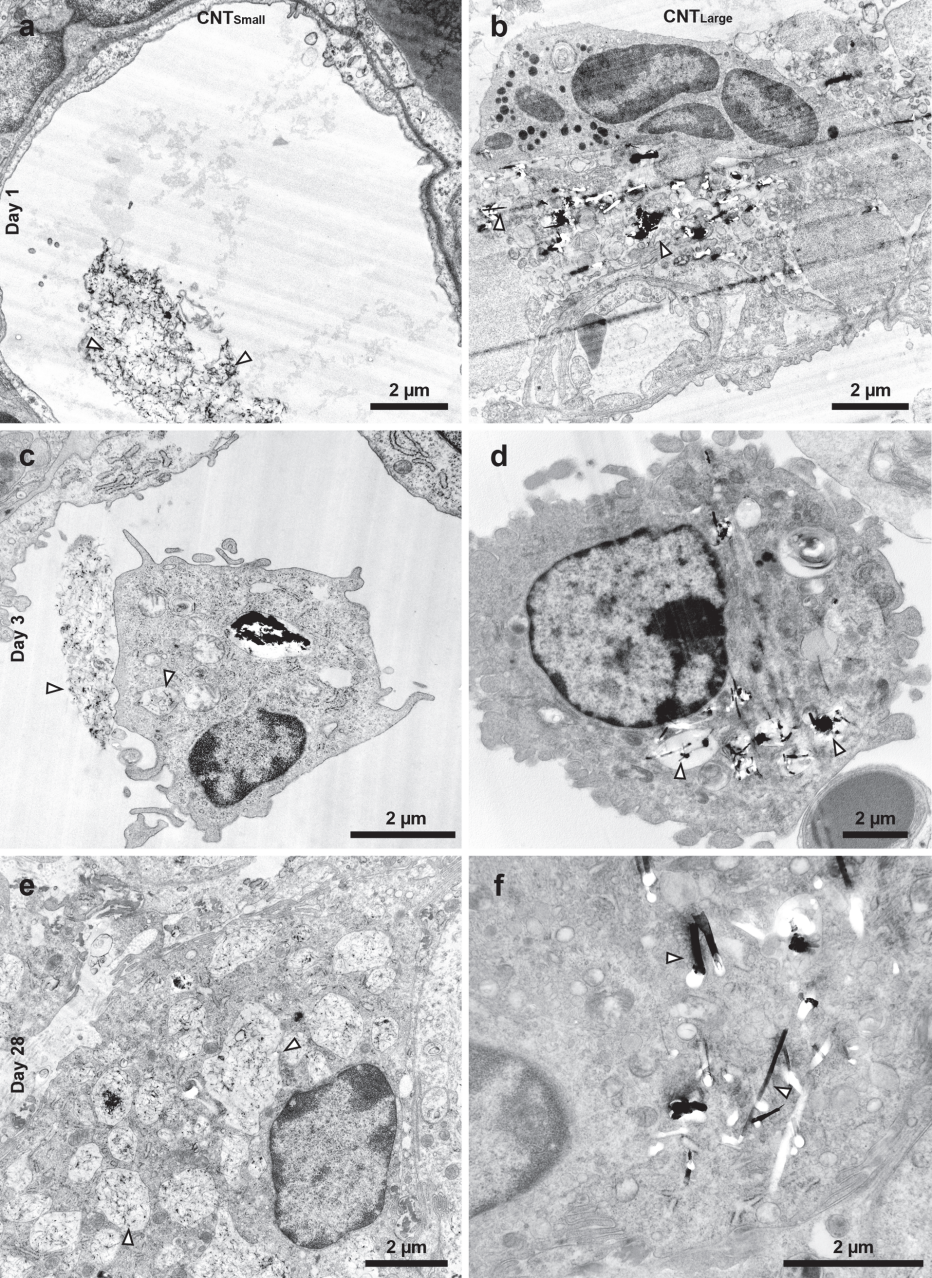
Representative images of the samples 1 day (a-b), 3 days (c-d), and 28 days (e-f) post exposure for CNT_Small_ (a,c,e) and CNT_Large_ (b,d,f). (a-b) Shows CNT_Small_ and CNT_Large_, respectively, on day 1, where CNTs were mainly found in the alveolar lumen or in the interface between cells. (c-d) On day 3 CNTs were increasingly found in vesicles inside what appeared to be alveolar macrophages, but some CNTs were observed outside cells. (e) On day 28, CNT_Small_ was found inside vesicles and in the cytosol of cells in large cell agglomerations, but were also found inside vesicles in singular cells. (f) On day 28, there were fewer vesicles containing CNT_Large_ and the CNTs were more individually spaced in the cytosol. In addition, immune cells were found in large agglomerations indicating inflammation at this stage. Arrowheads points to CNTs.

On post exposure day 1, minimal CNT-cell interaction was observed (Fig. 2 A-B). The CNTs were primarily aggregated outside cells in the alveolar lumen (Fig. 1 ‘between cells’). The individual CNTs were in close proximity to cells. In a few cases, CNTs were observed apparently in the process of entering the cell via physical indentation and subsequent piercing of the cellular membrane (Fig. 1 ‘piercing’). However, these observations were rare, and for CNT_Large_ and Mitsui-7 it was difficult to ascertain if cells were pierced due to ultramicrotomy artefacts caused by the large CNTs. We did not observe any clear signs of vesicular uptake at this time point, and only few immune cells were seen.

On post exposure day 3, we observed increasing cell-CNT interaction and increased numbers of what appeared to be inflammatory cells (Fig. 2 C-D), in agreement with the increased number of BAL cell counts observed at this time point. Large and small open cytoplasmic inclusions were observed containing agglomerated CNTs, apparently in the process of being taken up by the cells (Fig. 1 ‘inclusions’). Again, CNT_Small_ were observed directly entering the cytosol by direct piercing of the membrane, whereas this could not be verified for CNT_Large_ (Fig. 1 ‘piercing’). Three days post exposure, CNT_Small_ were primarily observed enclosed within vesicles (Fig. 1 ‘in vesicles’), and few CNTs were observed as free in the cytosol. CNT_Large_ uptake resulted in CNTs being enclosed in vesicles (Fig. 1 ‘in vesicles’), in addition to single CNTs found freely in the cytosol (Fig. 1 ‘in cytosol’). In a few cases, CNT_Large_ caused clear deformation of the vesicles inside alveolar macrophages, which was not observed to the same extent for CNT_Small_, and appeared to be involved in the process of vesicular escape (Fig. 1 ‘vesicular escape’).

There were fewer CNTs present 28 days post exposure compared to three days post exposure, and the CNTs were almost exclusively found inside cells and not in the alveolar lumen. CNTs were located within single alveolar macrophages and in larger inflammatory sites within multinucleate giant cells [30] (S1 Fig.). CNT_Small_ were found in both small and large vesicles with large amounts of CNTs inside some individual cells (Fig. 2 E). In certain cases, CNTs were found deforming vesicle membranes or between vesicles (Fig. 1 ‘vesicular escape’). CNT_Large_ was found inside deformed vesicles or sporadically distributed in the cytosol on day 28 (Fig. 2 F). Generally, there were fewer obvious CNT-containing vesicular structures compared to day 3 for CNT_Large_.

At all the studied time points, CNTs were found in alveolar macrophages, identified by their ultrastructure and location in the alveoli. We did not observe any CNTs in what appeared to be type II pneumocytes, but we observed a few CNT_Small_ that appeared to be inside what looked like type I pneumocytes. Likewise, CNTs were not observed inside well-defined neutrophils or eosinophils. Cells were identified and classified according to their morphological traits [31]. However, in some instances, especially in lung tissue on day 28, it was difficult to unambiguously determine the cell types of the cells containing the CNTs because the cell morphology was strongly perturbed by the CNTs (Fig. 2 E), and cells were found in agglomerates. CNTs were never observed inside the nucleus, except for what appeared to be caused by ultramicrotomy artefacts showing obvious drag marks. CNTs occasionally caused indentation of the nucleus, as previously observed *in vitro* for cells grown on nanowires [32,33].

### Eosinophilic crystalline pneumonia

**Figure 3:**
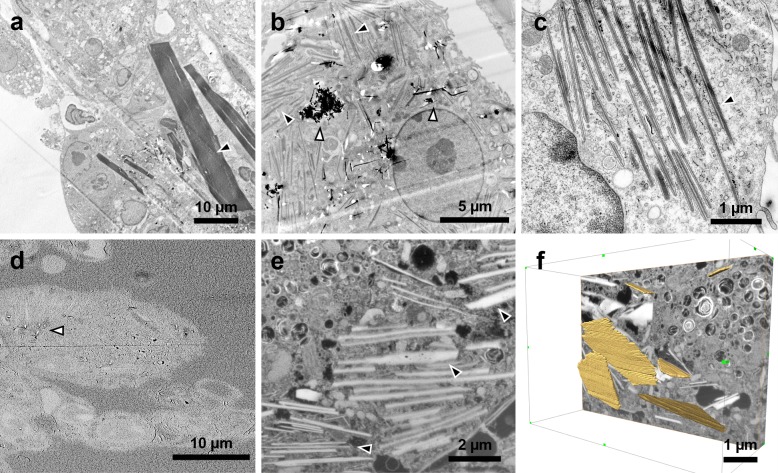
TEM (a-c) and SEM (d-f) images of crystalline bodies observed on day 28 in CNT_Large_ samples. (a) Low magnification TEM image showing large crystals, some up to 40 μm long. (b) Image showing scattered crystalline bodies and CNTs in clusters. (c) Ordered crystalline bodies in the cytosol. (d) SEM image of the ultramicrotomed block revealing the underlying cells prior to milling. The image also shows a few protruding CNTs (white arrowhead) [27]. (e) FIB-SEM image of crystals in a cell. (f) 3D representation of the FIB-SEM stack where a few of the crystals have been traced (yellow), revealing their plate-like structure. Black arrowheads indicate ECP crystals while white arrowheads indicate the CNTs.

TEM images revealed crystalline bodies in the cytosol of alveolar macrophages (Fig. 3 A-C) and multinucleated-cells (S1 Fig.) in the CNT_Large_ and Mitsui-7 exposed mice. Similar crystal structures have been observed in mice with eosinophilic crystalline pneumonia (ECP) [34–37]. Crystal bodies were observed in lung tissues from Mitsui-7 exposed mice for the highest dose on day 3, and in lung tissue from mice exposed to both the medium and high doses on day 28. For CNT_Large_ treated mice, crystalline bodies were observed on day 28 for the medium and high doses (S3 Table).

In some cases, the crystals appeared as needle-like structures similar in size to CNT_Large_ and Mitsui-7, but occasionally massive crystalline bodies up to 40 μm long and 5 μm wide were observed (Fig. 3 A). The structures were found in two variants: sharply defined enclosed in vesicles, and more diffusely defined enclosed in vesicles (S2 Fig.). ECP crystals were also observed outside cells, as previously documented [35,36,38]. Some of the observed structures had periodic lattice structures in the longitudinal direction (about 5 nm), a feature previously described [37], and the structures were clearly not CNTs as they did not cause characteristic microtomy artefacts of CNTs [27].

In order to elucidate the three dimensional structure of the crystals, we examined a sample containing crystals with slice-and-view 3D FIB-SEM (Fig. 3 D-F). In single slices the crystals were very similar to those observed in TEM with an inverted contrast (Fig. 3 E), but assembled crystals appeared as orthorhombic plates that often were stacked and piled up (Fig. 3 F and S1 Movie).

**Figure 4:**
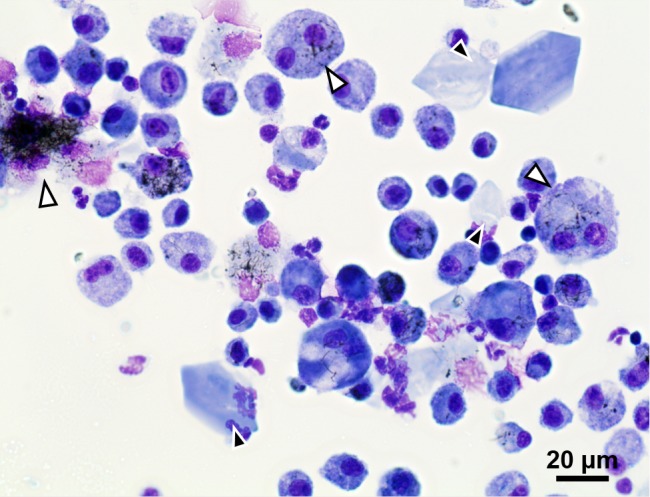
Representative image of stained eosinophilic crystals in BAL from mice exposed to the highest dose (162 μg) of CNT_Large_ 28 days post exposure. Large amounts of crystals were also observed for the two lower doses (18 μg and 54 μg), and similarly for Mitsui-7 samples on day 28. Black arrowheads point to crystals, whereas white arrowheads indicate CNT agglomerations or cells containing CNTs.

BAL cells were examined in light microscopy to confirm the presence of extracellular crystals related to ECP [36,38]. There was an increased crystal incidence in the CNT_Large_ and Mitsui-7 exposed samples on day 28. This was not observed to the same degree in CNT_Small_ exposed samples or the control samples (Table 1). In addition, the crystals found in CNT_Small_ and control samples were fewer in number and smaller compared to the crystals observed in CNT_Large_ and Mitsui-7 exposed samples. Generally, the crystals varied vastly in size and were mostly stained blue or had a blue periphery (Fig. 4 and S2 Fig.). In BAL slides from exposed mice, CNTs were observed inside alveolar macrophages and in agglomerations apparently outside of the cells (Fig. 4). No CNTs were observed in vehicle controls.

After the discovery of crystals in the present study, BAL cells previously recovered from mice 28 days after intratracheal instillation with similar doses of carbon black [39], and two single walled carbon nanotubes (SWCNT) [40], were also screened for the presence of crystals. 10% (2/17) of carbon black (Printex 90) samples contained crystals, whereas the two SWCNTs had a 50% crystal incidence in the 18 samples investigated per SWCNT (S3 Table).

### Gene expression

Eosinophilic crystals consist of the protein Chi3L3 encoded by *Chi3L3* [38,41,42]. Chi3L3 protein, also known as Ym1 and ECF-L, accumulates in alveolar macrophages and is associated with inflammatory diseases and parasite infestations [41,42]. We therefore assessed *Chi3L3* mRNA expression using available global gene expression data. Detailed description of the global gene expression analysis of lung tissues following pulmonary exposure to Mitsui-7 has been published [16], and will be published elsewhere for CNT_Small_ and CNT_Large_ (Poulsen *et al*. in peer review).

Both Mitsui-7 and CNT_Large_ induced statistically significant increases in *Chi3L3* mRNA levels whereas no change in *Chi3L3* expression was observed following CNT_Small_ exposure (Table 1). *Chi3L3* expression was increased on post-exposure day 1 and 3 following CNT_Large_ exposure for the two lower doses, whereas a significant increase was observed for the highest dose on day 28 (Table 1), coinciding with the observation of ECP crystals on day 28. Similar observations were made following Mitsui-7 exposure (Table 1).

In addition to the increased expression of *Chi3L3*, the expression of several chemokines and cytokines specific to eosinophil activity were increased following exposure to all three CNTs (S4 Table). Eosinophil migration and influx into the lung lumen is highly dependent on CCR3, a chemokine receptor that is abundant on eosinophils [43]. The chemokines CCL11 (eotaxin 1) and CCL24 (eotaxin 2) are produced by epithelial cells, smooth muscle cells and macrophages, and they have a high affinity for CCR3 [43–46]. The expression of these chemokines varied greatly following exposure to Mitsui-7, CNT_Small_ and CNT_Large_ (S4 Table). *Ccl11* and *Ccl24* were differentially expressed only at the high dose on day 3 following exposure to CNT_Small_, whereas both chemokines were expressed continuously at all doses on day 1 and 3 following exposure to CNT_Large_ and Mitsui-7. This correlates with the higher eosinophil influx observed following CNT_Large_ and Mitsui-7 exposure, as compared to CNT_Small_.

## Discussion

**Figure 5:**
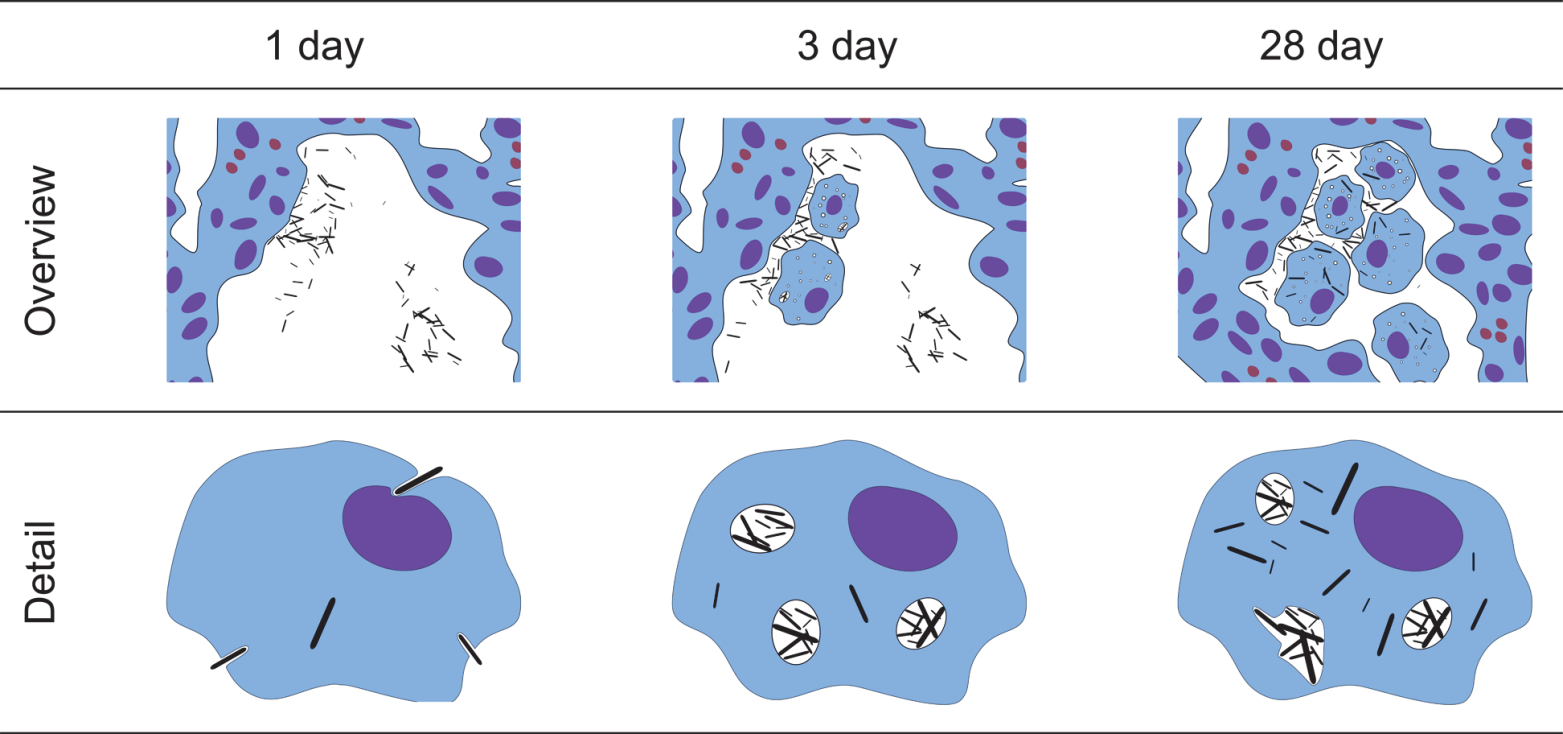
Model showing CNT interactions with lung tissue over time *in vivo*. At day 1, most CNTs were found in the alveolar spacing, while a few CNTs were found freely inside cells apparently having entered the cells using a non-endocytotic pathway. At day 3, CNTs are found in the alveolar spacing and a larger number of CNTs are found within vesicles, indicating an endocytotic-pathway. At day 28, most of the CNTs were either taken up by cells or in close relation to cells. Compared to post exposure day 3, the two larger CNTs had more CNTs outside vesicle bounds, indicating CNT escape from vesicles.

Cellular interactions of three different CNT were characterized at acute and intermediate time points. The observed cellular interactions of the three different CNTs were quite similar, with uptake following a general CNT distribution progression as illustrated in Fig. 5. The initial cell response on day 1 was dominated by CNTs situated in the alveoli, with only a few CNTs observed in the apparent process of piercing cellular membranes. Three days post exposure CNTs were observed as free in the cytosol and in intracellular vesicles, with a majority of CNTs being agglomerated in vesicles. On day 28, more individually dispersed CNTs were observed intracellularly for CNT_Large_ and Mitsui-7, whereas CNT_Small_ mainly were found in vesicles. In addition, deformed vesicles were observed, where CNTs deformed or penetrated the enclosing membrane. This was particularly evident for CNT_Large_ and Mitsui-7. This time-dependent progression agrees well with a previously proposed model for CNT uptake in cells based on *in vitro* experiments [24]. The model states that CNTs can be taken up into endosomes or by directly piercing through the cellular membrane. Subsequently, CNTs can escape vesicle enclosure, and in certain cases free CNTs in the cytosol can cross the nuclear membrane. We however only observed CNTs in the nucleus when ultramicrotomy artefacts were suspected [27].

The TEM studies revealed two different pathways by which CNTs may enter cells. First, the presence of agglomerates of CNTs in cellular inclusions indicates an endocytic and actively driven uptake mechanism [47]. Second, single CNTs traversing the cellular membrane may be indicative of physical piercing of the cell membrane, as also observed *in vitro* [7,23,25]. The ratio between CNTs taken up via physical piercing and via endocytosis was not quantified, but physical piercing was observed on day 1 whereas endocytic uptake was not. On day 3, CNTs were predominately found inside vesicles, suggesting a preceding endocytic uptake. It is unclear whether the CNTs are taken up by piercing and subsequently enter vesicles as some propose [12,24]. Nevertheless, the overall observed non-phagocytic uptake was small compared to the extensive phagocytic uptake seen on post exposure day 3 for all CNTs.

We found that CNTs appear to escape vesicle encapsulation over time *in vivo* as described previously in *in vitro* studies [23,24]. This is based on an observed decrease in the number of intracellular CNT-containing vesicles, the deformed vesicles observed on day 28, and also the higher level of cytosolic CNTs, particularly for CNT_Large_ and Mitsui-7 exposed samples, altogether indicating that CNTs can escape vesicles. The mechanism could be size related, as the observed CNT escape was less significant for CNT_Small_. In animal studies, CNTs similarly sized to CNT_Large_ have been observed freely in the cytosol of subpleural and alveolar macrophages weeks to months after exposure [21,22], whereas smaller and tangled CNTs have been found predominantly within phagosomes weeks [48] to months [49] after exposure. Thus, although the CNTs in the size range studied here did not incur frustrated phagocytosis, the longest of the studied CNTs may disrupt the endosomes and phagosomes and thereby cause single cell damage and be more difficult to clear. This may also explain why we see a more pronounced long-term toxicological effect for CNT_Large_ compared to CNT_Small_ in terms of pulmonary inflammation and cytokine expression.

### Eosinophilic crystalline pneumonia

Several mice in this study developed eosinophilic crystalline pneumonia (ECP). At first glance, the crystals appeared to be needle-like in the TEM sections (Fig. 3 A-C), whereas they in the light microscopy of BAL cell samples, were more cuboid or hexagonal (Fig. 4). 3D FIB-SEM imaging revealed that the needle-like structures in the TEM sections were caused by the sectioning, and that the crystals were in fact more plate-like and in the 3D imaging appeared to be similar to those observed in the BAL cell samples.

ECP is especially prevalent in the C57BL/6 mouse strain [34]. The incidence of ECP in aged female C57BL mice (10–30 months old) is approximately 16% [50], and generally younger mice develop ECP granulation less often than aged mice [42]. Therefore, the observed 10% incidence (2 out of 18) of ECP in the vehicle exposed mice on day 28 is in agreement with previous observations (Table 1). The more than 80% crystal incidence observed in both the CNT_Large_ and Mitsui-7 samples indicates a substantial increase (S3 Table). The 30–50% ECP incidence observed for CNT_Small_ and the additionally two SWCNTs BAL samples suggest smaller, but still increased ECP incidence. In contrast our BAL samples from carbon black nanoparticle exposure did not increase crystal incidence. This suggests that the increased development of ECP crystals may be related to the shape and dimensions of carbon-based particles.

In support of the crystals being related to ECP, we found increased gene expression of *Chi3L3* [38]. Increased *Chi3L3* expression on days 1, 3 or 28 coincided with increased incidence of crystals (Table 1). Increased Chi3L3 protein levels have previously been observed in lungs of mice exposed to SWCNT and asbestos [51], but the presence of ECP was not assessed.

### Eosinophilic response

The larger observed eosinophilic response to CNT_Large_ and Mitsui-7 than CNT_Small_ suggests that these larger CNTs cause a stronger allergy-like effect [17,52]. Eosinophilic inflammation is not a typical response following a single pulmonary exposure to particles [39,53,53,54].

The data suggests that ECP and eosinophilic response may correlate: The very large eosinophilic response on day 3 for the two larger CNTs (Table 1) correlated with the observation of crystals in BAL cell fluids on day 28. Likewise, the lower eosinophil response and *Chi3L3* expression levels of CNT_Small_, compared to CNT_Large_ and Mitsui-7, correlate with fewer ECP positive samples at post exposure day 28.

An eosinophilic response induced by MWCNTs has been observed in literature, but this has often been in studies using C57BL/6 or other ECP sensitive strains [10,16–18,55]. In contrast, studies noting an eosinophil influx using non-ECP sensitized strains as a response to CNT exposure are relatively few; e.g., experiments conducted on rats [19] or experiments involving allergens [52,56].

It has been reported that eosinophils are able to biodegrade CNTs [57], although they are normally thought to be involved in extracellular degradation of parasites which can be larger than the cell [57]. We speculate that the increased eosinophilic response, prevalent primarily in Mitsui-7 and CNT_Large_, might be due to long and thick CNTs being more difficult to internalize and degrade by alveolar macrophages. This correlates with the high extracellular CNT content on days 1 and 3 where the eosinophil influx was observed and the subsequent decrease in eosinophil number on day 28 when most tubes have been internalized.

## Conclusion

Our ultrastructural investigations revealed that all of the CNTs examined in this study appeared to follow the same overall progression over time: CNTs were taken up either by a diffusion mechanism or via endocytosis, CNTs agglomerated in vesicles, and lastly the CNTs appeared to escape vesicle enclosure. Our *in vivo* studies agree with *in vitro* studies [24] showing a similar overall sequence of events for uptake of MWCNTs.

TEM imaging further suggests that CNT_Large_ and Mitsui-7 were better able to perturb and escape vesicular enclosures in immune cells compared to CNT_Small_. The larger CNTs’ apparent ability to better escape vesicle enclosures, also correlate well with information gained by comparing multiple *in vivo* studies [21,22,48,49]. We speculate that comparably large and stiff CNTs (30–70 nm wide and 0.5–5 μm long) more frequently escape the endosomal system than smaller and more tangled CNTs.

The longer and thicker CNTs induced more ECP and BAL eosinophil influx, which was less pronounced for CNT_Small_ and SWCNTs. The ECP and eosinophil response appear correlated and given an ECP incidence in excess of 80% this could be an important factor to test in future studies.

## Supporting Information

S1 Table Physicochemical characterization of the studied carbon nanotubes. Mean CNT diameter and length were determined by TEM with the range describing the standard deviation (SD). The specific surface area (SSA) was determined by nitrogen adsorption using BET (Braunauer-Emmett-Teller). DLS describes the average aggregate size determined by Dynamic Light Scattering (DLS) of well-dispersed CNTs. Chemical composition was determined by wavelength dispersive X-ray fluorescence (WDXRF), results were manually post-processed for each individual element, to account for low concentration and peak overlaps, and data was calculated as wt% of the oxides of the elements.
*Data from the Nanogenotox joint action programme funded by EU Health Programme (2009 21 01).
^#^Data from Jackson et al. 2014 (submitted for publication elsewhere).
(DOCX)

S2 Table BAL cell count. Differential BAL cell counts (×10^3^) and SEM in the parentheses for the three CNTs. Values for vehicle instilled mice are based on N = 22–25, and N = 5–6 for CNT exposed mice. The control groups for CNT_Small_ and CNT_Large_ were pooled, since these were instilled using the same instillation vehicle (2% serum) and BAL cell composition of the control groups was not significantly different from each other. Mitsui-7 was instilled using a different vehicle (10% BAL in 0.9%NaCl), BAL cell composition of Mitsui-7 controls was statistically different from the other control groups. The Mitsui-7 exposure groups were therefore analysed separately. Statistically significantly different data (p < 0.05) from vehicle instilled mice are marked with an asterisk (*).
(DOCX)

S3 Table ECP overview. Overview of the *Chi3L3* level, eosinophil count (Eos) and ECP crystal positive samples using light microscopy (LM) or TEM (yes indicates positive findings, no negative findings). Dash (-) denotes no data available, while an asterisk (*) denotes statistically significant data (p<0.05). Thomas Swan and Sigma Long BAL cell slides were acquired from a previous study: Saber AT, Lamson JS, Jacobsen NR, et al. (2013) Particle-Induced Pulmonary Acute Phase Response Correlates with Neutrophil Influx Linking Inhaled Particles and Cardiovascular Risk. PLoS ONE 8:e69020. doi: 10.1371/journal.pone.0069020
(DOCX)

S4 Table *Ccl11* and *Ccl24* mRNA levels. *Ccl11* and *Ccl24* mRNA levels are given as the relative fold-increase in mRNA levels relative to concurrent vehicle controls, where values below 1 indicate a lowered mRNA level. Statistically significant changes (p<0.05) are marked with an asterisk (*).
(DOCX)

S1 Fig Multinucleate cells. Multinucleate cells as observed in light microscopy of BAL cells (CNT_Large_, 54 μg, day 3) and transmission electron microscopy images of tissue (CNT_Large_, 162 μg, day 28).
(TIF)

S2 Fig Eosinophilic crystal variation. Light microscopy images of the eosinophilic crystals in BAL fluid (a-c). TEM images of eosinophilic crystals in the lung tissue (d-f). The crystals varied greatly in size (a), and also in how well they were stained (b-c). (d) Crystals in TEM were observed intracellularly as sharply defined or with more blurred edges, and were also found extracellularly (e). (f) Shows a periodic ∼5 nm structure in a crystal.
(TIF)

S1 Movie 3D FIB-SEM video of ECP crystals. The image volume is 16.6 × 12.5 × 5.8 μm large, and crystals have manually been traced in the individual slides.
(AVI)

## References

[1] Monthioux M, Kuznetsov VL (2006) Who should be given the credit for the discovery of carbon nanotubes? Carbon 44: 1621–1623. 10.1016/j.carbon.2006.03.019.

[2] Donaldson K, Poland CA, Murphy FA, MacFarlane M, Chernova T, et al. (2013) Pulmonary toxicity of carbon nanotubes and asbestos—Similarities and differences. Advanced Drug Delivery Reviews 65: 2078–2086. 10.1016/j.addr.2013.07.014 23899865

[3] Pauluhn J (2009) Subchronic 13-week inhalation exposure of rats to multiwalled carbon nanotubes: Toxic effects are determined by density of agglomerate structures, not fibrillar structures. Toxicological Sciences 113: 226–242. 10.1093/toxsci/kfp247 19822600

[4] Aschberger K, Johnston HJ, Stone V, Aitken RJ, Hankin SM, et al. (2010) Review of carbon nanotubes toxicity and exposure—Appraisal of human health risk assessment based on open literature. Critical Reviews in Toxicology 40: 759–790. 10.3109/10408444.2010.506638 20860524

[5] Porter DW, Hubbs AF, Mercer RR, Wu N, Wolfarth MG, et al. (2010) Mouse pulmonary dose—and time course-responses induced by exposure to multi-walled carbon nanotubes. Toxicology 269: 136–147. 10.1016/j.tox.2009.10.017 19857541

[6] Hamilton RF, Wu Z, Mitra S, Shaw PK, Holian A (2013) Effect of MWCNT size, carboxylation, and purification on in vitro and in vivo toxicity, inflammation and lung pathology. Particle and Fibre Toxicology 10: 57 10.1186/1743-8977-10-57 24225053 PMC3830505

[7] Nagai H, Okazaki Y, Chew SH, Misawa N, Yamashita Y, et al. (2011) Diameter and rigidity of multiwalled carbon nanotubes are critical factors in mesothelial injury and carcinogenesis. Proceedings of the National Academy of Sciences 108: E1330–E1338. 10.1073/pnas.1110013108 22084097 PMC3241783

[8] Poland CA, Duffin R, Kinloch I, Maynard A, Wallace WAH, et al. (2008) Carbon nanotubes introduced into the abdominal cavity of mice show asbestos-like pathogenicity in a pilot study. Nature Nanotech 3: 423–428. 10.1038/nnano.2008.111 18654567

[9] Muller J, Huaux F, Fonseca A, Nagy JB, Moreau N, et al. (2008) Structural defects play a major role in the acute lung toxicity of multiwall carbon nanotubes: Toxicological aspects. Chemical Research in Toxicology 21: 1698–1705. 10.1021/tx800101p 18636756

[10] Wang X, Xia T, Addo Ntim S, Ji Z, Lin S, et al. (2011) Dispersal state of multiwalled carbon nanotubes elicits profibrogenic cellular responses that correlate with fibrogenesis biomarkers and fibrosis in the murine lung. ACS Nano 5: 9772–9787. 10.1021/nn2033055 22047207 PMC4136431

[11] Ali-Boucetta H, Nunes A, Sainz R, Herrero MA, Tian B, et al. (2013) Asbestos-like pathogenicity of long carbon nanotubes alleviated by chemical functionalization. Angewandte Chemie 125: 2330–2334. 10.1002/ange.201207664. 23319294

[12] Lacerda L, Russier J, Pastorin G, Herrero MA, Venturelli E, et al. (2012) Translocation mechanisms of chemically functionalised carbon nanotubes across plasma membranes. Biomaterials 33: 3334–3343. 10.1016/j.biomaterials.2012.01.024 22289266

[13] Sager TM, Wolfarth MW, Andrew M, Hubbs A, Friend S, et al. (2014) Effect of multi-walled carbon nanotube surface modification on bioactivity in the C57BL/6 mouse model. Nanotoxicology 8: 317–327. 10.3109/17435390.2013.779757 23432020 PMC4669410

[14] Kapralov AA, Feng WH, Amoscato AA, Yanamala N, Balasubramanian K, et al. (2012) Adsorption of surfactant lipids by single-walled carbon nanotubes in mouse lung upon pharyngeal aspiration. ACS Nano 6: 4147–4156. 10.1021/nn300626q 22463369 PMC3358590

[15] Ma-Hock L, Treumann S, Strauss V, Brill S, Luizi F, et al. (2009) Inhalation toxicity of multiwall carbon nanotubes in rats exposed for 3 months. Toxicological Sciences 112: 468–481. 10.1093/toxsci/kfp146 19584127

[16] Poulsen SS, Jacobsen NR, Labib S, Wu D, Husain M, et al. (2013) Transcriptomic analysis reveals novel mechanistic insight into murine biological responses to multi-walled carbon nanotubes in lungs and cultured lung epithelial cells. PLoS ONE 8: e80452 10.1371/journal.pone.0080452 24260392 PMC3834097

[17] Erdely A, Liston A, Salmen-Muniz R, Hulderman T, Young S-H, et al. (2011) Identification of systemic markers from a pulmonary carbon nanotube exposure: Journal of Occupational and Environmental Medicine 53: S80–S86. 10.1097/JOM.0b013e31821ad724 21654424

[18] Girtsman TA, Beamer CA, Wu N, Buford M, Holian A (2014) IL-1R signalling is critical for regulation of multi-walled carbon nanotubes-induced acute lung inflammation in C57Bl/6 mice. Nanotoxicology 8: 17–27. 10.3109/17435390.2012.744110 23094697 PMC4080682

[19] Muller J, Huaux F, Moreau N, Misson P, Heilier J-F, et al. (2005) Respiratory toxicity of multi-wall carbon nanotubes. Toxicology and Applied Pharmacology 207: 221–231. 10.1016/j.taap.2005.01.008 16129115

[20] Ronzani C, Spiegelhalter C, Vonesch J-L, Lebeau L, Pons F (2012) Lung deposition and toxicological responses evoked by multi-walled carbon nanotubes dispersed in a synthetic lung surfactant in the mouse. Archives of Toxicology 86: 137–149. 10.1007/s00204-011-0741-y 21805258

[21] Ryman-Rasmussen JP, Cesta MF, Brody AR, Shipley-Phillips JK, Everitt JI, et al. (2009) Inhaled carbon nanotubes reach the subpleural tissue in mice. Nature Nanotechnology 4: 747–751. 10.1038/nnano.2009.305 19893520 PMC2783215

[22] Kobayashi N, Naya M, Ema M, Endoh S, Maru J, et al. (2010) Biological response and morphological assessment of individually dispersed multi-wall carbon nanotubes in the lung after intratracheal instillation in rats. Toxicology 276: 143–153. 10.1016/j.tox.2010.07.021 20696199

[23] Al-Jamal KT, Nerl H, Müller KH, Ali-Boucetta H, Li S, et al. (2011) Cellular uptake mechanisms of functionalised multi-walled carbon nanotubes by 3D electron tomography imaging. Nanoscale 3: 2627 10.1039/c1nr10080g 21603701

[24] Mu Q, Broughton DL, Yan B (2009) Endosomal leakage and nuclear translocation of multiwalled carbon nanotubes: Developing a model for cell uptake. Nano Letters 9: 4370–4375. 10.1021/nl902647x 19902917 PMC2796686

[25] Pantarotto D, Singh R, McCarthy D, Erhardt M, Briand J-P, et al. (2004) Functionalized carbon nanotubes for plasmid DNA gene delivery. Angewandte Chemie International Edition 43: 5242–5246. 10.1002/anie.200460437 15455428

[26] Marangon I, Boggetto N, Ménard-Moyon C, Venturelli E, Béoutis M-L, et al. (2012) Intercellular carbon nanotube translocation assessed by flow cytometry imaging. Nano Letters 12: 4830–4837. 10.1021/nl302273p 22928721

[27] Købler C, Saber AT, Jacobsen NR, Wallin H, Vogel U, et al. (2014) FIB-SEM imaging of carbon nanotubes in mouse lung tissue. Analytical and Bioanalytical Chemistry. Available: http://link.springer.com/10.1007/s00216-013-7566-x. Accessed 2014 Feb 26. 10.1007/s00216-013-7566-x PMC4039996 24448971

[28] Nanogenotox (2013) Towards a method for detecting the potential genotoxicity of nanomaterials. Available: www.nanogenotox.eu. Accessed 2014 Apr 28.

[29] Porter DW, Hubbs AF, Chen BT, McKinney W, Mercer RR, et al. (2013) Acute pulmonary dose–responses to inhaled multi-walled carbon nanotubes. Nanotoxicology 7: 1179–1194. 10.3109/17435390.2012.719649 22881873 PMC4687396

[30] Bhattacharya K, Andón FT, El-Sayed R, Fadeel B (2013) Mechanisms of carbon nanotube-induced toxicity: Focus on pulmonary inflammation. Advanced Drug Delivery Reviews 65: 2087–2097. 10.1016/j.addr.2013.05.012 23751779

[31] Ross MH (2011) Histology: a text and atlas: with correlated cell and molecular biology. 6th ed. Philadelphia: Wolters Kluwer/Lippincott Williams & Wilkins Health. 974 p.

[32] Persson H, Købler C, Mølhave K, Samuelson L, Tegenfeldt JO, et al. (2013) Fibroblasts cultured on nanowires exhibit low motility, impaired cell Division, and DNA Damage. Small 9: 4006–4016. 10.1002/smll.201300644 23813871 PMC4282547

[33] Wierzbicki R, Købler C, Jensen MRB, Łopacińska J, Schmidt MS, et al. (2013) Mapping the complex morphology of cell interactions with nanowire substrates using FIB-SEM. PLoS ONE 8: e53307 10.1371/journal.pone.0053307 23326412 PMC3541134

[34] Elmore SA, Berridge BR, Boyle MC, Cora MC, Hoenerhoff MJ, et al. (2012) Proceedings of the 2012 national toxicology program satellite symposium. Toxicologic Pathology 41: 151–180. 10.1177/0192623312467102 23262640 PMC4195569

[35] Feldmesser M, Kress Y, Casadevall A (2001) Intracellular crystal formation as a mechanism of cytotoxicity in murine pulmonary Cryptococcus neoformans infection. Infection and Immunity 69: 2723–2727. 10.1128/IAI.69.4.2723-2727.2001 11254641 PMC98213

[36] Guo L (2000) Biochemical characterization of endogenously formed eosinophilic crystals in the lungs of mice. Journal of Biological Chemistry 275: 8032–8037. 10.1074/jbc.275.11.8032 10713123

[37] Murray AB, Luz A (1990) Acidophilic macrophage pneumonia in laboratory mice. Vet Pathol 27: 274–281. 2169666 10.1177/030098589002700409

[38] Hoenerhoff MJ, Starost MF, Ward JM (2006) Eosinophilic crystalline pneumonia as a major cause of death in 129S4/SvJae mice. Veterinary Pathology 43: 682–688. 10.1354/vp.43-5-682 16966445

[39] Bourdon JA, Saber AT, Jacobsen NR, Jensen KA, Madsen AM, et al. (2012) Carbon black nanoparticle instillation induces sustained inflammation and genotoxicity in mouse lung and liver. Particle and Fibre Toxicology 9: 5 10.1186/1743-8977-9-5 22300514 PMC3293019

[40] Saber AT, Lamson JS, Jacobsen NR, Ravn-Haren G, Hougaard KS, et al. (2013) Particle-induced pulmonary acute phase response correlates with neutrophil influx linking inhaled particles and cardiovascular risk. PLoS ONE 8: e69020 10.1371/journal.pone.0069020 23894396 PMC3722244

[41] Nio J, Fujimoto W, Konno A, Kon Y, Owhashi M, et al. (2004) Cellular expression of murine Ym1 and Ym2, chitinase family proteins, as revealed by in situ hybridization and immunohistochemistry. Histochemistry and Cell Biology 121 Available: http://link.springer.com/10.1007/s00418-004-0654-4. Accessed 2013 Oct 4. 10.1007/s00418-004-0654-4 15148607

[42] Waern I, Jia J, Pejler G, Zcharia E, Vlodavsky I, et al. (2010) Accumulation of Ym1 and formation of intracellular crystalline bodies in alveolar macrophages lacking heparanase. Molecular Immunology 47: 1467–1475. 10.1016/j.molimm.2010.02.004 20226534

[43] Humbles AA, Lu B, Friend DS, Okinaga S, Lora J, et al. (2002) The murine CCR3 receptor regulates both the role of eosinophils and mast cells in allergen-induced airway inflammation and hyperresponsiveness. Proceedings of the National Academy of Sciences 99: 1479–1484. 10.1073/pnas.261462598 11830666 PMC122216

[44] Guo R-F, Lentsch AB, Warner RL, Huber-Lang M, Sarma JV, et al. (2001) Regulatory effects of eotaxin on acute lung inflammatory injury. The Journal of Immunology 166: 5208–5218. 11290805 10.4049/jimmunol.166.8.5208

[45] Heath H, Qin S, Rao P, Wu L, LaRosa G, et al. (1997) Chemokine receptor usage by human eosinophils. The importance of CCR3 demonstrated using an antagonistic monoclonal antibody. Journal of Clinical Investigation 99: 178–184. 10.1172/JCI119145 9005985 PMC507784

[46] Ben-Yehuda C, Bader R, Puxeddu I, Levi-Schaffer F, Breuer R, et al. (2008) Airway eosinophil accumulation and eotaxin-2/CCL24 expression following allergen challenge in BALB/c mice. Experimental Lung Research 34: 467–479. 10.1080/01902140802220625 18850374

[47] Canton I, Battaglia G (2012) Endocytosis at the nanoscale. Chemical Society Reviews 41: 2718 10.1039/c2cs15309b 22389111

[48] Wang X, Zang JJ, Wang H, Nie H, Wang TC, et al. (2010) Pulmonary toxicity in mice exposed to low and medium doses of water-soluble multi-walled carbon nanotubes. Journal of Nanoscience and Nanotechnology 10: 8516–8526. 10.1166/jnn.2010.2688 21121361

[49] Treumann S, Ma-Hock L, Groters S, Landsiedel R, van Ravenzwaay B (2013) Additional histopathologic examination of the lungs from a 3-month inhalation toxicity study with multiwall carbon nanotubes in rats. Toxicological Sciences 134: 103–110. 10.1093/toxsci/kft089 23570993

[50] Van Zweiten M., Zurcher C, Solleveld HA, Hollander CF (1981) Immunological techniques applied to aging research. Boca Raton, Fla: CRC Press. 236 p.

[51] Teeguarden JG, Webb-Robertson B-J, Waters KM, Murray AR, Kisin ER, et al. (2010) Comparative proteomics and pulmonary toxicity of instilled single-walled carbon nanotubes, crocidolite asbestos, and ultrafine carbon black in mice. Toxicological Sciences 120: 123–135. 10.1093/toxsci/kfq363 21135415 PMC3044201

[52] Nygaard UC, Hansen JS, Samuelsen M, Alberg T, Marioara CD, et al. (2009) Single-walled and multi-walled carbon nanotubes promote allergic immune responses in mice. Toxicological Sciences 109: 113–123. 10.1093/toxsci/kfp057 19293371

[53] Saber AT, Jacobsen N, Mortensen A, Szarek J, Jackson P, et al. (2012) Nanotitanium dioxide toxicity in mouse lung is reduced in sanding dust from paint. Particle and Fibre Toxicology 9: 4 10.1186/1743-8977-9-4 22300483 PMC3298479

[54] Saber AT, Jensen KA, Jacobsen NR, Birkedal R, Mikkelsen L, et al. (2012) Inflammatory and genotoxic effects of nanoparticles designed for inclusion in paints and lacquers. Nanotoxicology 6: 453–471. 10.3109/17435390.2011.587900 21649461

[55] Beamer CA, Girtsman TA, Seaver BP, Finsaas KJ, Migliaccio CT, et al. (2013) IL-33 mediates multi-walled carbon nanotube (MWCNT)-induced airway hyper-reactivity via the mobilization of innate helper cells in the lung. Nanotoxicology 7: 1070–1081. 10.3109/17435390.2012.702230 22686327 PMC4080677

[56] Inoue K, Koike E, Yanagisawa R, Hirano S, Nishikawa M, et al. (2009) Effects of multi-walled carbon nanotubes on a murine allergic airway inflammation model. Toxicology and Applied Pharmacology 237: 306–316. 10.1016/j.taap.2009.04.003 19371758

[57] Andón FT, Kapralov AA, Yanamala N, Feng W, Baygan A, et al. (2013) Biodegradation of single-walled carbon nanotubes by eosinophil peroxidase. Small 9: 2721–2729. 10.1002/smll.201202508 23447468 PMC4039041

