# Supplementary material for: Time-Dependent Subcellular Distribution and Effects of Carbon Nanotubes in Lungs of Mice
Source: PLoS One. 2015 Jan 23;10(1):e0116481. doi: 10.1371/journal.pone.0116481 (PMC4304811; doi:10.1371/journal.pone.0116481)
Supplement: S1 Table — Mean CNT diameter and length were determined by TEM with the range describing the standard deviation (SD). The specific surface area (SSA) was determined by nitrogen adsorption using BET (Braunauer-Emmett-Teller). DLS describes the average aggregate size determined by Dynamic Light Scattering (DLS) of well-dispersed CNTs. Chemical composition was determined by wavelength dispersive X-ray fluorescence (WDXRF), results were manually post-processed for each individual element, to account for low concentration and peak overlaps, and data was calculated as wt% of the oxides of the elements. *Data from the Nanogenotox joint action programme funded by EU Health Programme (2009 21 01). #Data from Jackson et al. 2014 (submitted for publication elsewhere). (DOCX) [file pone.0116481.s001.docx]

# Table S1: Physicochemical characterisation of the studied carbon nanotube

|  | CNT_Small_ | CNT_Large_ | Mitsui-7 |
| --- | --- | --- | --- |
| Nanogenotox code | NRCWE-026 | NM-401 | NRCWE-006 |
| Producer | Confidential | IO-LE-TECNanomaterials | Mitsui |
| Type | Pristine | Pristine | Pristine |
| Mean length^*^ (± SD) | 0.85±0.46 µm | 4.05 ± 2.37 µm | 5.73 ± 3.67 µm |
| Mean width^*^ (± SD) | 11±4 nm | 67 ± 35 nm | 74 ± 28 nm |
| Minimal width^*^ | 4 nm | 24 nm | 29 nm |
| Maximal width^*^ | 23 nm | 138 nm | 173 nm |
| BET SSA^*^ | 246 m^2^/g | 18 m^2^/g | 22 m^2^/g |
| Impurities^#^ | 15.6 wt% | 0.3 wt% | 0.4 wt% |
| Major impurities^#^ (wt%) | Al_2_O_3_ (14.97) | P_2_O_5_ (0.14) | P_2_O_5_ (0.14) |
|  | Fe_2_O_3_ (0.29) | SO_3_ (0.08) | SO_3_ (0.08) |
|  | CoO (0.11) | Fe_2_O_3_ (0.05) | Fe_2_O_3_ (0.08) |
|  | CaO (0.01) | CaO (0.03) | CaO (0.03) |
|  | Cl (0.01) | MgO (0.01) | MgO (0.01) |

Mean CNT diameter and length were determined by TEM with the range describing the standard deviation (SD). The specific surface area (SSA) was determined by nitrogen adsorption using BET (Braunauer-Emmett-Teller). DLS describes the average aggregate size determined by Dynamic Light Scattering (DLS) of well-dispersed CNTs. Chemical composition was determined by wavelength dispersive X-ray fluorescence (WDXRF), results were manually post-processed for each individual element, to account for low concentration and peak overlaps, and data was calculated as wt% of the oxides of the elements.

^*^Data from the Nanogenotox joint action programme funded by EU Health Programme (2009 21 01).

^#^Data from Jackson et al. 2014 (submitted for publication elsewhere).
