# Supplementary material for: Time-Dependent Subcellular Distribution and Effects of Carbon Nanotubes in Lungs of Mice
Source: PLoS One. 2015 Jan 23;10(1):e0116481. doi: 10.1371/journal.pone.0116481 (PMC4304811; doi:10.1371/journal.pone.0116481)
Supplement: S2 Table — Differential BAL cell counts (×103) and SEM in the parentheses for the three CNTs. Values for vehicle instilled mice are based on N = 22–25, and N = 5–6 for CNT exposed mice. The control groups for CNTSmall and CNTLarge were pooled, since these were instilled using the same instillation vehicle (2% serum) and BAL cell composition of the control groups was not significantly different from each other. Mitsui-7 was instilled using a different vehicle (10% BAL in 0.9%NaCl), BAL cell composition of Mitsui-7 controls was statistically different from the other control groups. The Mitsui-7 exposure groups were therefore analysed separately. Statistically significantly different data (p < 0.05) from vehicle instilled mice are marked with an asterisk (*). (DOCX) [file pone.0116481.s002.docx]

# Table S2: BAL cell composition following pulmonary instillation of MWCNTs.

Differential BAL cell counts (x10^3^) and SEM in the parentheses for the three CNTs. Values for vehicle instilled mice are based on N = 22-25, and N =5-6 for CNT exposed mice. The control groups for CNT_Small_ and CNT_Large_ were pooled, since these were instilled using the same instillation vehicle (2% serum) and BAL cell composition of the control groups was not significantly different from each other. Mitsui-7 was instilled using a different vehicle (10% BAL in 0.9%NaCl), and BAL cell composition of Mitsui-7 controls was statistically different from the other control groups. The Mitsui-7 exposure groups were therefore analysed separately. Statistically significantly different data (p < 0.05) from vehicle instilled mice are marked with an asterisk (*).

|  |  |  | **CNT_Small_** | | | **CNT_Large_** | | |  | **Mitsui-7** | | |
| --- | --- | --- | --- | --- | --- | --- | --- | --- | --- | --- | --- | --- |
|  |  | 0 µg | 18 µg | 54 µg | 162 µg | 18 µg | 54 µg | 162 µg | 0 µg | 18 µg | 54 µg | 162 µg |
| **Day 1** | Neutrophils | 9.4 | 54.7^*^ | 116.5^*^ | 89.5^*^ | 49.9^*^ | 94.1^*^ | 118.0^*^ | 7.7 | 55.1^*^ | 60.6^*^ | 140.2^*^ |
|  |  | (2.5) | (10.1) | (55.7) | (39.9) | (6.3) | (2.9) | (15.5) | (1.7) | (10.6) | (4.5) | (31.9) |
|  | Macrophages | 62.8 | 49.9 | 33.3^*^ | 22.5^*^ | 45.7 | 46.5^*^ | 15.4^*^ | 53.2 | 38.2 | 37.4 | 30.3^*^ |
|  |  | (5.9) | (9.2) | (5.3) | (4.4) | (9.4) | (4.9) | (2.9) | (2.5) | (5.1) | (7.4) | (8.5) |
|  | Eosinophils | 1.0 | 17.2^*^ | 1.4^*^ | 3.4^*^ | 50.6^*^ | 86.2^*^ | 1.7 | 0.3 | 38.8^*^ | 23.1^*^ | 2.2^*^ |
|  |  | (0.5) | (6.2) | (0.4) | (1.5) | (11.7) | (15.0) | (0.7) | (0.1) | (11.7) | (11.1) | (0.5) |
|  | Lymphocytes | 0.8 | 1.1 | 0.9 | 0.5 | 2.1 | 2.5 | 1.7 | 1.6 | 2.8 | 1.7 | 0.6^*^ |
|  |  | (0.2) | (0.5) | (0.4) | (0.3) | (0.3) | (0.7) | (0.7) | (0.5) | (0.5) | (0.5) | (0.3) |
|  | Total | 82.8 | 127.0^*^ | 160.0^*^ | 123.5 | 156.5^*^ | 240.5^*^ | 154.0 | 73.8 | 142.8^*^ | 134.7^*^ | 195.3^*^ |
|  |  | (8.3) | (11.1) | (53.2) | (45.8) | (11.6) | (14.9) | (20.1) | (3.6) | (22.3) | (19.5) | (40.8) |
| **Day 3** | Neutrophils | 1.7 | 109.8^*^ | 163.0^*^ | 458.6^*^ | 36.1^*^ | 101.3^*^ | 159.7^*^ | 3.0 | 84.3^*^ | 154.9^*^ | 163.2^*^ |
|  |  | (0.7) | (13.5) | (16.3) | (83.5) | (8.8) | (33.0) | (33.9) | (0.2) | (13.6) | (34.3) | (49.9) |
|  | Macrophages | 62.4 | 120.0 | 190.6^*^ | 37.5 | 72.7 | 79.7^*^ | 56.5 | 56.4 | 103.1^*^ | 119.7^*^ | 80.6 |
|  |  | (4.2) | (26.7) | (22.6) | (10.9) | (11.1) | (11.7) | (8.5) | (4.2) | (9.7) | (15.9) | (18.5) |
|  | Eosinophils | 3.9 | 69.2^*^ | 72.2^*^ | 7.1 | 317.3^*^ | 138.3^*^ | 1.8 | 0.4 | 341.3^*^ | 268.0^*^ | 100.9^*^ |
|  |  | (4.2) | (11.1) | (12.8) | (2.7) | (37.4) | (33.3) | (0.5) | (0.6) | (122.7) | (94.0) | (57.6) |
|  | Lymphocytes | 1.4 | 9.9^*^ | 19.5^*^ | 2.8 | 19.7^*^ | 16.9^*^ | 2.4 | 0.9 | 27.0^*^ | 42.4^*^ | 16.5^*^ |
|  |  | (0.4) | (3.7) | (5.9) | (1.0) | (6.2) | (3.6) | (0.8) | (0.2) | (9.7) | (10.2) | (5.9) |
|  | Total | 78.1 | 328.5^*^ | 473.0^*^ | 520.5^*^ | 465.0^*^ | 347.0^*^ | 231.5^*^ | 69.2 | 581.0^*^ | 610.3^*^ | 378.3^*^ |
|  |  | (5.7) | (30.2) | (16.2) | (87.4) | (43.6) | (43.6) | (41.8) | (6.4) | (121.0) | (148.1) | (75.5) |
| **Day 28** | Neutrophils | 3.8 | 29.0^*^ | 28.9^*^ | 38.0^*^ | 26.1^*^ | 17.6^*^ | 81.2^*^ | 1.1 | 10.2^*^ | 30.2^*^ | 90.4^*^ |
|  |  | (2.1) | (8.9) | (12.0) | (7.5) | (11.0) | (5.1) | (15.6) | (0.3) | (2.4) | (7.5) | (14.2) |
|  | Macrophages | 57.3 | 76.0 | 73.1 | 141.7^*^ | 76.2 | 78.3 | 86.5^*^ | 82.4 | 87.5 | 99.8 | 97.5 |
|  |  | (3.6) | (16.8) | (23.4) | (18.1) | (9.3) | (15.8) | (18.6) | (5.7) | (8.2) | (14.3) | (7.8) |
|  | Eosinophils | 10.5 | 0.5 | 0.2 | 0.0 | 33.5 | 32.3 | 46.3 | 0.3 | 5.5^*^ | 21.7^*^ | 45.5^*^ |
|  |  | (5.4) | (0.2) | (0.2) | (0.0) | (13.2) | (14.2) | (17.5) | (0.0) | (1.8) | (8.5) | (13.6) |
|  | Lymphocytes | 3.0 | 16.6^*^ | 7.1^*^ | 9.2^*^ | 14.9^*^ | 19.9^*^ | 9.5^*^ | 2.1 | 16.8^*^ | 23.1^*^ | 45.8^*^ |
|  |  | (0.9) | (6.0) | (3.3) | (2.0) | (3.6) | (8.2) | (2.3) | (0.4) | (6.2) | (5.8) | (9.3) |
|  | Total | 84.4 | 137.0^*^ | 122.0 | 206.0^*^ | 167.0^*^ | 159.5 | 240.5^*^ | 95.9 | 133.2 | 189.1^*^ | 293.3^*^ |
|  |  | (9.2) | (23.6) | (38.4) | (28.5) | (34.7) | (35.5) | (41.2) | (5.8) | (16.3) | (31.6) | (34.4) |
