# Supplementary material for: Time-Dependent Subcellular Distribution and Effects of Carbon Nanotubes in Lungs of Mice
Source: PLoS One. 2015 Jan 23;10(1):e0116481. doi: 10.1371/journal.pone.0116481 (PMC4304811; doi:10.1371/journal.pone.0116481)
Supplement: S3 Table — Overview of the Chi3L3 level, eosinophil count (Eos) and ECP crystal positive samples using light microscopy (LM) or TEM (yes indicates positive findings, no negative findings). Dash (-) denotes no data available, while an asterisk (*) denotes statistically significant data (p<0.05). Thomas Swan and Sigma Long BAL cell slides were acquired from a previous study: Saber AT, Lamson JS, Jacobsen NR, et al. (2013) Particle-Induced Pulmonary Acute Phase Response Correlates with Neutrophil Influx Linking Inhaled Particles and Cardiovascular Risk. PLoS ONE 8:e69020. doi: 10.1371/journal.pone.0069020 (DOCX) [file pone.0116481.s003.docx]

# Table S3: ECP overview

Overview of the *Chi3L3* level, eosinophil count (Eos) and ECP crystal positive samples using light microscopy (LM) or TEM (yes indicates positive findings, no negative findings). Dash (-) denotes no data available, while an asterix (*) denotes statistically significant data (p<0.05).

|  |  | **Day 1** | | | | **Day 3** | | | | **Day 28** | | | |
| --- | --- | --- | --- | --- | --- | --- | --- | --- | --- | --- | --- | --- | --- |
| ***Type*** | ***Dose [µg]*** | ***Chi3L3*** | ***Eos*** | ***ECP*** | | ***Chi3L3*** | ***Eos*** | ***ECP*** | | ***Chi3L3*** | ***Eos*** | ***ECP*** | |
|  |  |  |  | ***LM*** | ***TEM*** |  |  | ***LM*** | ***TEM*** |  |  | ***LM*** | ***TEM*** |
| CNT_Small_ | 0 | - | 1 | - | No | - | 2.4 | - | No | - | 0.6 | 0/6 | No |
|  | 18 | 2.0 | 17.2^*^ | 0/1 | - | 1.4 | 69.2^*^ | 0/2 | - | 1.0 | 0.5 | 2/6 | No |
|  | 54 | 1.0 | 1.4 | 0/1 | - | 0.5 | 72.2^*^ | 0/2 | - | 0.9 | 0.2 | 1/6 | No |
|  | 162 | 1.0 | 3.4 | 0/1 | No | 0.7 | 7.1 | 0/2 | No | 1.7 | 0.0 | 3/6 | No |
| CNT_Large_ | 0 | - | 1 | - | - | - | 5.3 | - | - | - | 20.4 | 1/6 | - |
|  | 18 | 2.8^*^ | 50.6^*^ | - | - | 5.8^*^ | 317.3^*^ | 0/2 | - | 1.8 | 33.5 | 6/6 | No |
|  | 54 | 3.1^*^ | 86.2^*^ | - | - | 4.3^*^ | 138.3^*^ | 1/2 | - | 2.5 | 32.3 | 5/6 | Yes |
|  | 162 | 1.1 | 1.7 | - | No | 1.1 | 1.8 | 0/2 | No | 9.2^*^ | 46.3 | 4/6 | Yes |
| Mitsui7 | 0 | - | 0.3 | - | - | - | 0.4 | - | - | - | 0.3 | 1/6 | No |
|  | 18 | - | 38.8^*^ | - | - | - | 341.3^*^ | 0/2 | - | - | 5.5 | 6/6 | - |
|  | 54 | - | 23.1^*^ | - | - | - | 268.0^*^ | 0/2 | - | - | 21.7^*^ | 6/6 | Yes |
|  | 162 | - | 2.2^*^ | - | No | - | 100.9 | 0/2 | Yes | - | 45.5^*^ | 6/6 | Yes |
| Carbon Black  (Printex 90) | 18 | - | - | - | - | - | - | - | - | - | - | 1/6 |  |
|  | 54 | - | - | - | - | - | - | - | - | - | - | 1/6 |  |
|  | 162 | - | - | - | - | - | - | - | - | - | - | 0/5 |  |
| SWCNT1  (Thomas Swan) | 18 | - | - | - | - | - | - | - | - | - | - | 2/6 |  |
|  | 54 | - | - | - | - | - | - | - | - | - | - | 3/3 |  |
|  | 162 | - | - | - | - | - | - | - | - | - | - | 4/6 |  |
| SWCNT2  (Sigma Long) | 18 | - | - | - | - | - | - | - | - | - | - | 5/6 |  |
|  | 54 | - | - | - | - | - | - | - | - | - | - | 3/6 |  |
|  | 162 | - | - | - | - | - | - | - | - | - | - | 3/6 |  |

Thomas Swan and Sigma Long BAL cell slides were acquired from a previous study: Saber AT, Lamson JS, Jacobsen NR, et al. (2013) Particle-Induced Pulmonary Acute Phase Response Correlates with Neutrophil Influx Linking Inhaled Particles and Cardiovascular Risk. PLoS ONE 8:e69020. doi: 10.1371/journal.pone.0069020
