# Supplementary material for: Time-Dependent Subcellular Distribution and Effects of Carbon Nanotubes in Lungs of Mice
Source: PLoS One. 2015 Jan 23;10(1):e0116481. doi: 10.1371/journal.pone.0116481 (PMC4304811; doi:10.1371/journal.pone.0116481)
Supplement: S4 Table — Ccl11 and Ccl24 mRNA levels are given as the relative fold-increase in mRNA levels relative to concurrent vehicle controls, where values below 1 indicate a lowered mRNA level. Statistically significant changes (p<0.05) are marked with an asterisk (*). (DOCX) [file pone.0116481.s004.docx]

# Table S4: *Ccl11* and *Ccl24* mRNA levels

*CCL11* and *CCL24* mRNA levels normalized to levels in concurrent controls. Values below 1 indicate lowered mRNA levels. Statistically significant changes (p<0.05) are marked with an asterix (*)

| **Mitsui-7** | | | |  | **CNT_Small_** | | | |  | **CNT_Large_** | | | |
| --- | --- | --- | --- | --- | --- | --- | --- | --- | --- | --- | --- | --- | --- |
| Ccl11 | | | |  | Ccl11 | | | |  | Ccl11 | | | |
|  | **18 µg** | **54 µg** | **162 µg** |  |  | **18 µg** | **54 µg** | **162 µg** |  |  | **18 µg** | **54 µg** | **162 µg** |
| **Day 1** | 3.0^*^ | 5.2^*^ | 3.4^*^ |  | **Day 1** | 3.3 | 2.2 | 2.2 |  | **Day 1** | 5.6^*^ | 9.3^*^ | 4.3^*^ |
| **Day 3** | - | - | - |  | **Day 3** | 1.2 | 2.9 | 7.1^*^ |  | **Day 3** | 5.4^*^ | 7.4^*^ | 3.9^*^ |
| **Day 28** | - | 1.5 | - |  | **Day 28** | 1.6 | 1.0 | 2.6 |  | **Day 28** | 1.5 | 1.5 | 3.6 |
| Ccl24 | | | |  | Ccl24 | | | |  | Ccl24 | | | |
|  | **18 µg** | **54 µg** | **162 µg** |  |  | **18 µg** | **54 µg** | **162 µg** |  |  | **18 µg** | **54 µg** | **162 µg** |
| **Day 1** | 10.7^*^ | 9.8^*^ | 2.7 |  | **Day 1** | 3.6 | 1.2 | 0.8 |  | **Day 1** | 6.1 | 20.6 | 2.3 |
| **Day 3** | - | - | - |  | **Day 3** | 1.2 | 2.3 | 8.5^*^ |  | **Day 3** | 11.7^*^ | 21.5^*^ | 4.6^*^ |
| **Day 28** | - | 1.6 | - |  | **Day 28** | 0.7 | 1.1 | 0.8 |  | **Day 28** | 0.9 | 0.8 | 2.4 |
