# Supplementary figures and images for: Time-Dependent Subcellular Distribution and Effects of Carbon Nanotubes in Lungs of Mice
Source: PLoS One. 2015 Jan 23;10(1):e0116481. doi: 10.1371/journal.pone.0116481 (PMC4304811; doi:10.1371/journal.pone.0116481)

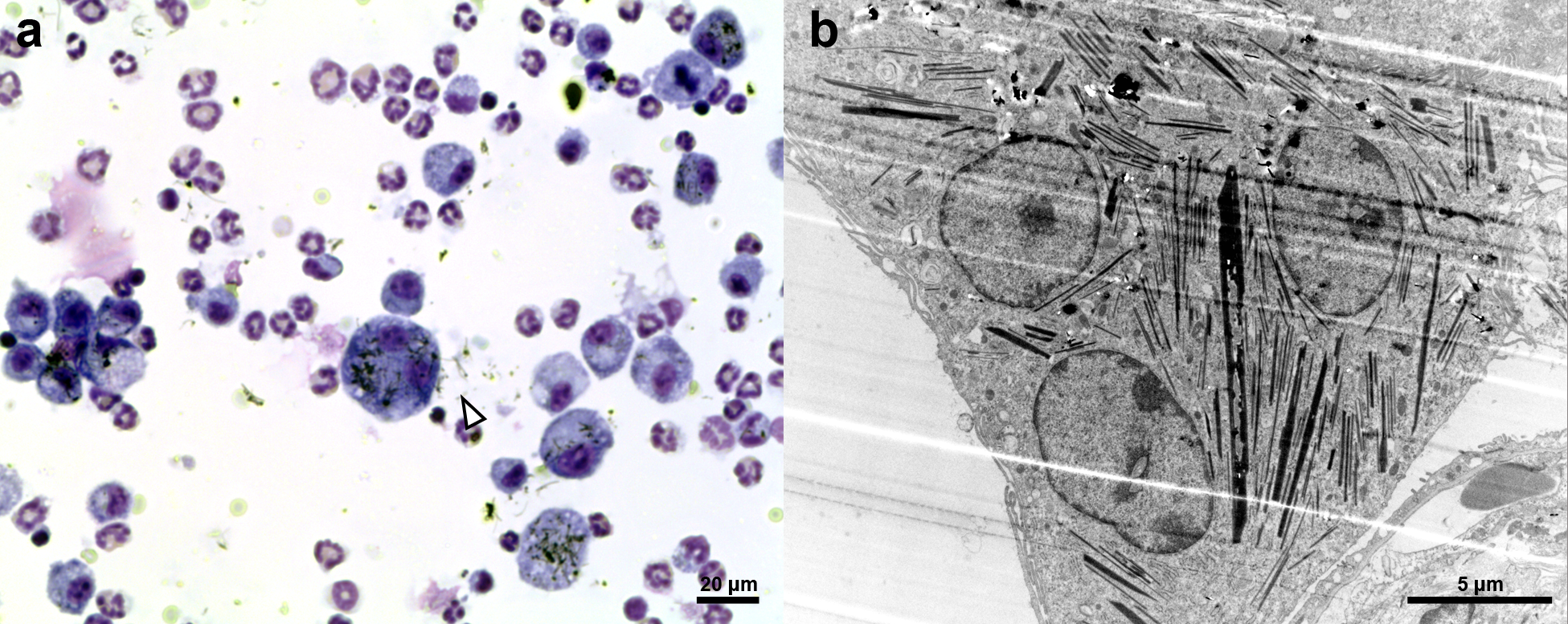

Supplement: S1 Fig — Multinucleate cells as observed in light microscopy of BAL cells (CNTLarge, 54 μg, day 3) and transmission electron microscopy images of tissue (CNTLarge, 162 μg, day 28). (TIF) [file pone.0116481.s005.tif]

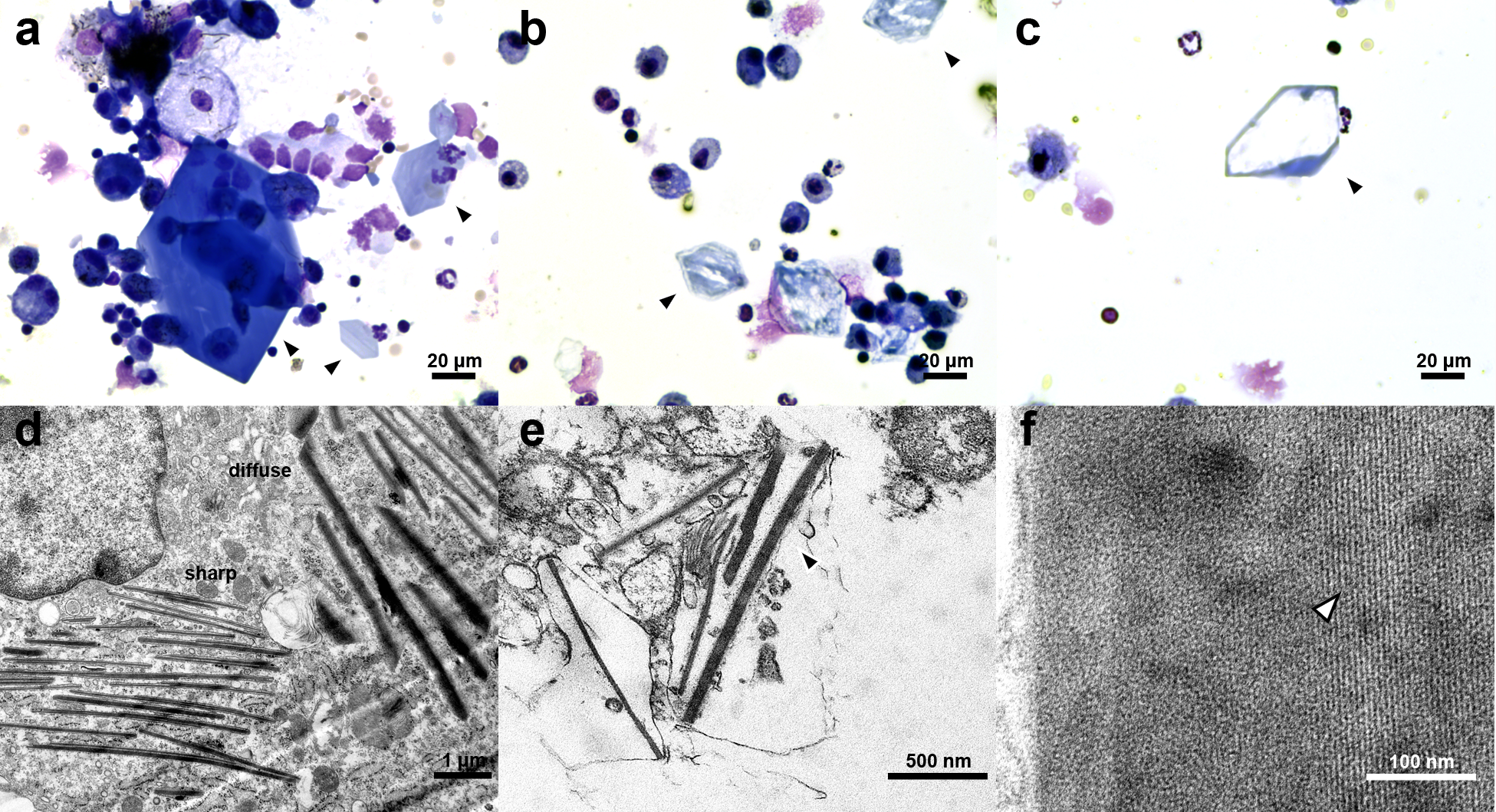

Supplement: S2 Fig — Light microscopy images of the eosinophilic crystals in BAL fluid (a-c). TEM images of eosinophilic crystals in the lung tissue (d-f). The crystals varied greatly in size (a), and also in how well they were stained (b-c). (d) Crystals in TEM were observed intracellularly as sharply defined or with more blurred edges, and were also found extracellularly (e). (f) Shows a periodic ∼5 nm structure in a crystal. (TIF) [file pone.0116481.s006.tif]
